# An endogenous microRNA (miRNA1166.1) can regulate photobio-H_2_ production in eukaryotic green alga *Chlamydomonas reinhardtii*

**DOI:** 10.1186/s13068-018-1126-8

**Published:** 2018-05-02

**Authors:** Yuting Wang, Xiaoshan Zhuang, Meirong Chen, Zhiyong Zeng, Xiaoqi Cai, Hui Li, Zhangli Hu

**Affiliations:** 1 Guangdong Technology Research Center for Marine Algal Bioengineering, Guangdong Key Laboratory of Plant Epigenetic, College of Life Sciences and Oceanography, Shenzhen University, Shenzhen, 518060 People’s Republic of China; 2 Shenzhen Key Laboratory of Marine Bioresource & Eco-environmental Science, Longhua Innovation Institute for Biotechnology, College of Life Sciences and Oceanography, Shenzhen University, Shenzhen, 518060 People’s Republic of China

**Keywords:** Biohydrogen, Green alga, *Chlamydomonas reinhardtii*, microRNA, Gene regulation

## Abstract

**Background:**

Hydrogen photoproduction from green microalgae is regarded as a promising alternative solution for energy problems. However, the simultaneous oxygen evolution from microalgae can prevent continuous hydrogen production due to the hypersensitivity of hydrogenases to oxygen. Sulfur deprivation can extend the duration of algal hydrogen production, but it is uneconomical to alternately culture algal cells in sulfur-sufficient and sulfur-deprived media.

**Results:**

In this study, we developed a novel way to simulate sulfur-deprivation treatment while constantly maintaining microalgal cells in sulfur-sufficient culture medium by overexpressing an endogenous microRNA (miR1166.1). Based on our previous RNA-seq analysis in the model green alga *Chlamydomonas reinhardtii*, three endogenous miRNAs responsive to sulfur deprivation (cre-miR1166.1, cre-miR1150.3, and cre-miR1158) were selected. Heat-inducible expression vectors containing the selected miRNAs were constructed and transformed into *C. reinhardtii*. Comparison of H_2_ production following heat induction in the three transgenic strains and untransformed control group identified miR1166.1 as the best candidate for H_2_ production regulation. Moreover, enhanced photobio-H_2_ production was observed with repeated induction of miR1166.1 expression.

**Conclusions:**

This study is the first to identify a physiological function of endogenous miR1166.1 and to show that a natural miRNA can regulate hydrogen photoproduction in the unicellular model organism *C. reinhardtii*.

**Electronic supplementary material:**

The online version of this article (10.1186/s13068-018-1126-8) contains supplementary material, which is available to authorized users.

## Background

Hydrogen gas (H_2_) is a clean energy source, and hydrogen production from green algae is regarded as a promising alternative solution for energy problems, since green algae have iron hydrogenases with high enzyme activity and can rely on solar energy for growth [1–8]. However, hydrogen photoproduction in green algae lasts for just a few seconds to a few minutes due to the hypersensitivity of iron hydrogenases to simultaneously generate oxygen from photosynthesis. Moreover, algal cells only produce hydrogen with excess electrons from the photosynthetic chain to prevent overreduction [9–12]; thus, the critical problem restricting the application of hydrogen production by green algae is the unsustainability of hydrogen biosynthesis.

A conventional sustainable hydrogen production method depends on the reversible inactivation of oxygen evolution through two-stage algal cultivation [13–15]. The first stage is oxygenic photosynthesis to accumulate algal biomass, and the second stage is anaerobic hydrogen production in a sulfur-deprived medium. Under sulfur deprivation, algal cell respiration exhausts oxygen and causes anaerobiosis in the culture. Therefore, this two-stage method separates hydrogen production from oxygen evolution and carbon accumulation, and sulfur deprivation makes sustained hydrogen production possible [11]. However, the replacement of normal medium with sulfur-deprived medium requires the centrifugation of algal cells, which may affect cell activity and is difficult to apply in large-scale production.

Comparative studies of sulfur-deprived and sulfur-replete algae have been conducted at different molecular levels in *Chlamydomonas reinhardtii* [13–20]. A better understanding of the molecular mechanism of sulfur-deprived stress response may pave new ways for genetic regulation to improve algal photobio-H_2_ production. Previous results showed that sulfur-deprived cells had massive changes in cellular physiology and metabolism, along with the accumulation of proteins with fewer sulfur-containing amino acids [17]. Microarray analyses showed that photosynthetic genes, one exception being *LHCBM9*, are generally repressed by sulfur deprivation, suggesting a major remodeling of the photosystem II light-harvesting complex [16]. Sulfur deprivation also affects proteins related to photosynthetic machinery, protein biosynthetic apparatuses, molecular chaperones, and 20S proteasomal components [17]. Recently, we found that non-coding RNAs, including microRNAs (miRNAs), which function as important gene regulators, are also significantly influenced by sulfur deprivation [18, 19]. Further analysis suggested that miRNAs are potentially involved in photobio-H_2_ production in *C. reinhardtii*.

miRNAs are a class of endogenous non-coding small RNAs in eukaryotes. They are approximately 18–24 nucleotide (nt) in length and processed from RNAs into stem–loop precursor structures by the nuclease Dicer. miRNAs regulate target gene expression by promoting mRNA degradation and/or inhibiting protein translation. Since miRNAs are present in the unicellular green alga *C. reinhardtii*, the miRNA pathway represents an ancient gene regulation mechanism that evolved prior to the emergence of multicellularity [21, 22]. In multicellular organisms, miRNAs have been shown to be an important and widespread regulator. Animal miRNAs are involved in developmental timing, cell death, cell proliferation, and patterning of the nervous system [23, 24]. In fact, about one-third of human genes may be miRNA targets [25]. miRNAs are associated with the development of diseases including cancer [26, 27], and they regulate a range of developmental and physiological processes, such as hematopoiesis [28]. In plants, miRNAs also play dominant roles in post-transcriptional gene regulation and modulate organ development, phase transition, and stress responses [29]. In contrast, the functions of miRNAs in unicellular eukaryotic algae remain largely unknown [30]. In *C. reinhardtii*, some miRNAs (Cre-miR914 and Cre-miR910) were found related to multiple stresses (heat shock, UV-B, and salinity) [31], but functions of most miRNAs still haven’t been studied.

In this study, three endogenous miRNAs responsive to sulfur deprivation (cre-miR1166.1, cre-miR1150.3, and cre-miR1158) were selected from a previous RNA-seq database in the green alga *C. reinhardtii*. Heat-inducible miRNA overexpression transformants were constructed. By comparing H_2_ production following heat induction in the three transgenic strains and the WT group, miR1166.1 was identified as the best candidate for H_2_ production regulation. Moreover, the enhanced photobio-H_2_ production of miR1166.1-overexpressing algae could be repeatedly induced. To our knowledge, this is the first report of an endogenous miRNA affecting photobio-H_2_ production in *C. reinhardtii*.

## Methods

### Algal strains and culture conditions

The cell-wall-deficient *C. reinhardtii* strain CC-849 served as the receptor strain and negative control. Algal cells were cultured in TAP (Tris–acetate-phosphate) medium at 22 °C under continuous cool-white light. Sulfur-deprivation medium (TAP-S) was prepared by replacing the S-salts with their chloride counterparts. For 1 L of TAP-S medium, 40× Filner’s Beijernicks Solution (25 mL), 1 M potassium phosphate (1 mL), trace mineral solution (1 mL), and Tris base (2.42 g) were combined, and the pH was adjusted to 7.0 with glacial acetic acid. For sulfur-deprivation treatment, 400 mL cells at exponential phase were collected by centrifugation then washed twice with liquid TAP-S medium. Equal quantities of algal cells were resuspended in TAP and TAP-S media and grown for up to 72 h under continuous white light illumination (≈ 20 μmol photons m^−2^ s^−1^). The cells were collected for RNA isolation, and the sulfate concentration in the supernatant was detected using a Dionex ICS-1100 ion chromatograph [4, 15].

### Sequencing of miRNA

Total RNA was extracted using Trizol reagent (Invitrogen) according to the manufacturer’s protocol, and RNA quality was examined on an Agilent 2100 Bioanalyzer. For library construction, small RNAs were purified by PAGE and ligated to adaptors. Sequencing of the two libraries was performed using Illumina Solexa sequencing. Raw reads were filtered and compared to the miRBase database (release 15.0). Further details can be found in our previous work [18].

### Construction of miRNA overexpression vectors

The pH124 vector, which contains the *ble* gene for zeocin resistance, was used as the expression vector [32–35]. An artificial miRNA precursor was introduced into pH124 to permit inducibility by high heat shock treatment in *C. reinhardtii*. The artificial miRNA precursor was based on the backbone of the highly expressed cre-miR1162 precursor [36–38]. The constructed artificial miRNA precursor sequences were synthesized in vitro, and the *Nhe*I (GCTAGC) and *Pma*CI (CACGTG) digestion sites were used to insert the sequences into pH124. The construct containing the promoter, miRNA precursor, and *ble* gene integrated randomly into the alga nuclear genome.

### Genomic DNA PCR analysis

PCR was performed to verify the transformation of the artificial miRNA precursor constructs into algal cells. PCR was performed using the primer pair 593-F (5′-TGACCTCCACTTTCAGCGACA-3′) and 593-R (5′-ACTTGAGAGCAGTATCTTCCATCCA-3′), which map to the region outside of the multiple cloning site in the pH124 vector. The PCR cycling conditions were as follows: pre-denaturation at 94 °C for 4 min; 30 amplification cycles of 94 °C for 30 s, 60 °C for 30 s, and 72 °C for 60 s; and a final extension for 10 min at 72 °C. All amplification products were verified by sequencing and alignment analysis.

### Hydrogen detection

Equivalent culture volumes of CC-849 and the transgenic algal strains (400 mL) were cultured in 500 ml bottles sealed with rubber sheet septa until exponential phase. The cultures were pre-treated in the dark for 24 h then cultivated under continuous light to detect gas contents. Gas samples (1 mL sample volume) from the headspace of the cultures were drawn with a syringe and separated using a molecular sieve column (length 6 ft, 1/8 in. OD, 2 mm ID, MolSieve 5A packing, mesh size 60/80). A gas chromatograph with a thermal conductivity detector was used to detect the concentration of H_2_ (Agilent 7890A; Agilent Technologies Inc., USA). Argon was used as the carrier gas [4]. To test the sustained hydrogen production capacity (Fig. 6), we used triplicates for each strain, and cultured them with the same initial cell density under the same condition. Algal cells at exponential phase were treated in 40 °C water incubates for 1 h and then recovered to 22 °C. After 3, 5, 7, and 9 h of recovery, these cells were heated for the second time and recovered for 3, 5, and 7 h before the heat and recovery were repeated for the third time. H_2_ concentrations were measured respectively at the mentioned times.

### Small RNA extraction

Small RNAs were isolated from both CC-849 and transgenic algae using the E.Z.N.A.^®^ miRNA Kit (OMEGA) according to the manufacturer’s protocol. Polyadenylation was performed using the Poly (A) Tailing Kit (Takara) according to manufacturer’s instructions. The small RNAs were reverse transcribed using poly (T) adapter (Additional file 1: Table S1) according to the S-Poly(T) method [39].

### Quantitative real-time PCR

Quantitative real-time PCR (qRT-PCR) was used to measure changes in miR1166.1 expression in CC-849 and transgenic algae. Quantitative real-time PCR was performed using SYBR Premix Ex TaqTM II (Takara, Japan) according to the manufacturer’s instructions on an Applied Biosystems 7300 Real-Time PCR System (Framingham, MA, USA). Quantitative real-time PCR was performed using a universal reverse primer and miR1166.1-specific and U4-specific forward primers [39]. The primer sequences are listed in Additional file 1: Table S1. The PCR cycling conditions were as follows: pre-denaturation at 95 °C for 30 s, followed by 40 two-step cycles of 95 °C for 10 s and 60 °C for 30 s. U4 snRNA was used as the reference gene for the quantitative real-time PCR detection of miR1166.1. The data were analyzed using the 2^−ΔΔCt^ calculation method.

## Results

### Identification of miRNAs potentially involved in *C. reinhardtii* photobio-H_2_ production

**Fig. 1:**
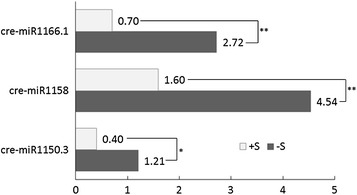
Normalized expression levels of sulfur-deprivation-responsive miRNAs in sulfur-replete (+S) and sulfur-deprived (−S) samples. The numbers beside the boxes indicate the normalized expression levels from high-throughput sequencing. **Indicates fold change (log2) > 1 and *p* < 0.01. *Indicates fold change (log2) > 1 and 0.01 ≤ *p* < 0.05. Normalized expression = actual miRNA count/total count of clean reads * 1000000

We previously performed high-throughput RNA sequencing to identify differentially expressed miRNAs in *C. reinhardtii* after sulfur-deprivation stress. The analysis identified 310 miRNAs, including 85 miRNAs in miRBase (http://www.mirbase.org/) and 225 novel miRNAs [18]. Among the 310 miRNAs, 47 miRNAs (24 known and 23 novel ones) were responsive to sulfur deprivation, and most of them were up-regulated. We chose three miRNAs that were significantly up-regulated after sulfur deprivation, cre-miR1166.1, cre-miR1150.3, and cre-miR1158, which were up-regulated by 3.9, 2.8, and 3.0 fold, respectively (Fig. 1).

### Construction of miRNA overexpression transgenic algae

**Fig. 2:**
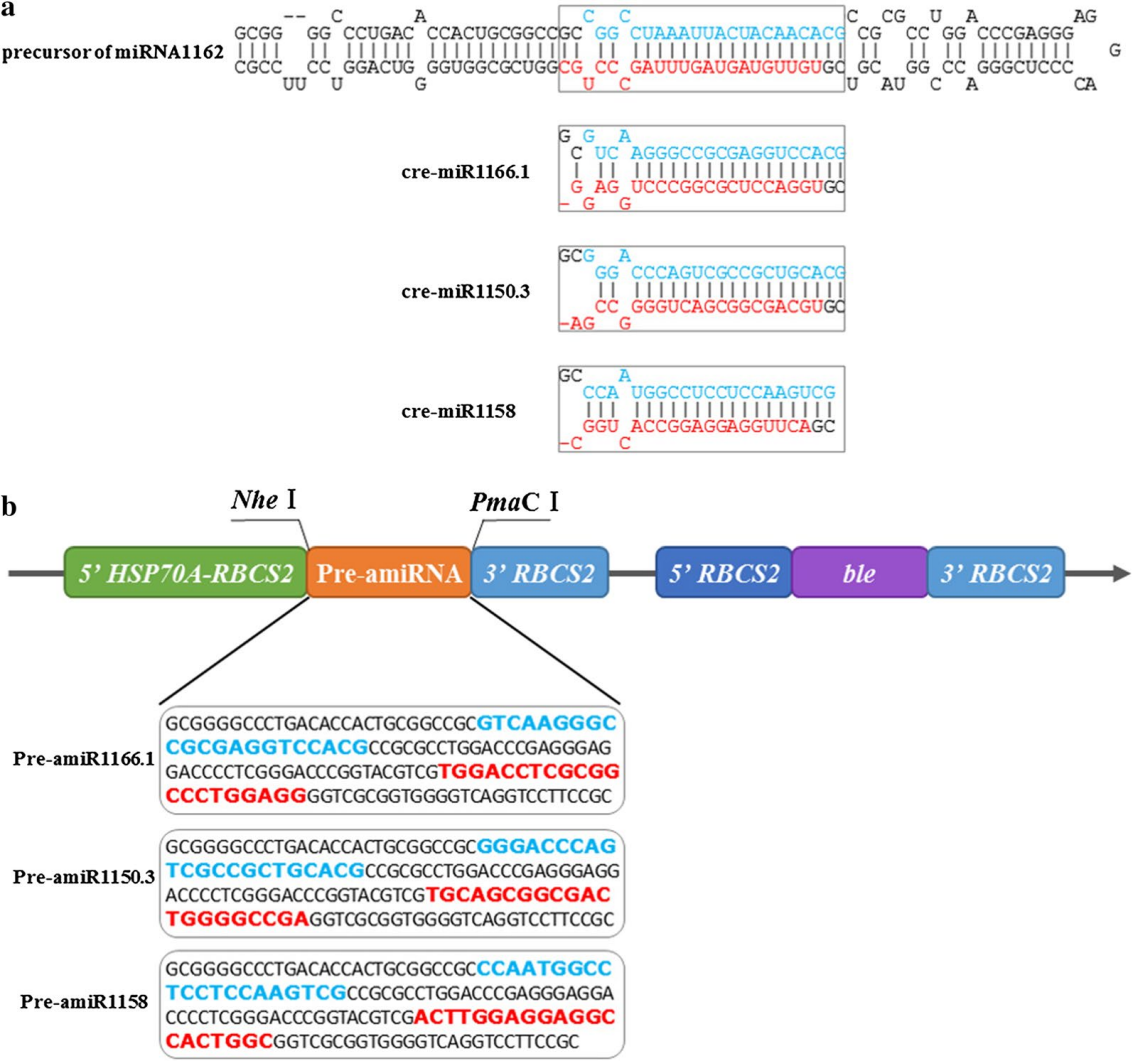
Construction of pre-miRNA vectors. The mature miRNA sequences of miR1166.1, miR1150.3, and miR1158 are shown in red, and the miRNA* sequences are shown in blue. **a** Stem–loop structures of the miR1162 precursor and the amiR1166.1, amiR1150.3, and amiR1158 precursors. The mature components (miRNA/miRNA* duplex) are shown in boxes. **b** Partial diagram of the pH124-miRNA expression vector. *Nhe*I and *Pma*CI sites were added to the left and right arms of the miR1162 precursor, respectively. 5′ *HSP70A* is a heat shock promoter, 5′ *RBCS2* is a constitutive promoter, and *ble* is the zeocin resistance gene

The expression vector pH124 harbors the *HSP70A*-*RBCS2* promoter upstream of the multiple cloning site [32] and is commonly used to drive exogenous gene expression upon heat induction in *C. reinhardtii* [4, 33–35]. The precursor of cre-miR1162, a highly expressed endogenous miRNA of *C. reinhardtii*, was used as the expression backbone [36], and the mature sequence of cre-miR1162 was replaced with that of cre-miR1166.1, cre-miR1150.3, and cre-miR1158 in our study (Fig. 2a). The three miRNA precursors were introduced into the heat-inducible expression vector pH124 (Fig. 2b), and the constructed vectors were transformed into the cell-wall-deficient *C. reinhardtii* strain CC-849. The transgenic algal strains, referred to as T-miR1166.1, T-miR1150.3, and T-miR1158, were screened with the antibiotic zeocin. Positive clones were confirmed by genomic DNA PCR, and the PCR products were sequenced and aligned (Additional file 2: Figure S1). The results showed that the mature miRNA sequences contained in the three vectors had been correctly integrated into the *C. reinhardtii* nuclear genome.

### Selection of high H_2_ production transgenic algal strains

**Fig. 3:**
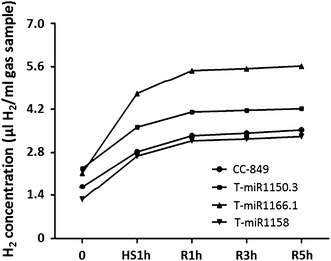
Selection of high H_2_ production transgenic algae. The *Y*-axis (H_2_ concentration) indicates the H_2_ content in 1 mL gas sample detected by gas chromatography (GC), which was calculated from the H_2_ peak area. *0* untreated controls; *HS1h* heat shock treatment for 1 h; *R1h, R3h, and R5h* recovery at 22 °C for 1, 3, and 5 h after heat treatment, respectively. Since H_2_ detection of each sample takes 7–8 min, biological replicates were performed for each transgenic algal strain to avoid large treatment time differences. Biological replicates are shown in Fig. 6, Additional file 3: Figure S2, and Additional file 4: Figure S3

H_2_ production levels were compared between the three transgenic algae and the untransformed CC-849 control by gas chromatography to investigate the effect of the overexpressed miRNAs. Compared to CC-849, transformants T-miR1166.1 and T-miR1150.3 exhibited enhanced H_2_-producing capacity, while the capacity of T-miR1158 was similar to the control. The effect in T-miR1166.1 exceeded that in T-miR1150.3, and H_2_ production in T-miR1166.1 was twofold greater compared to CC-849 (Fig. 3). Consequently, we chose miR1166.1 for further analysis.

### Characteristics and secondary structure of miR1166.1

**Fig. 4:**
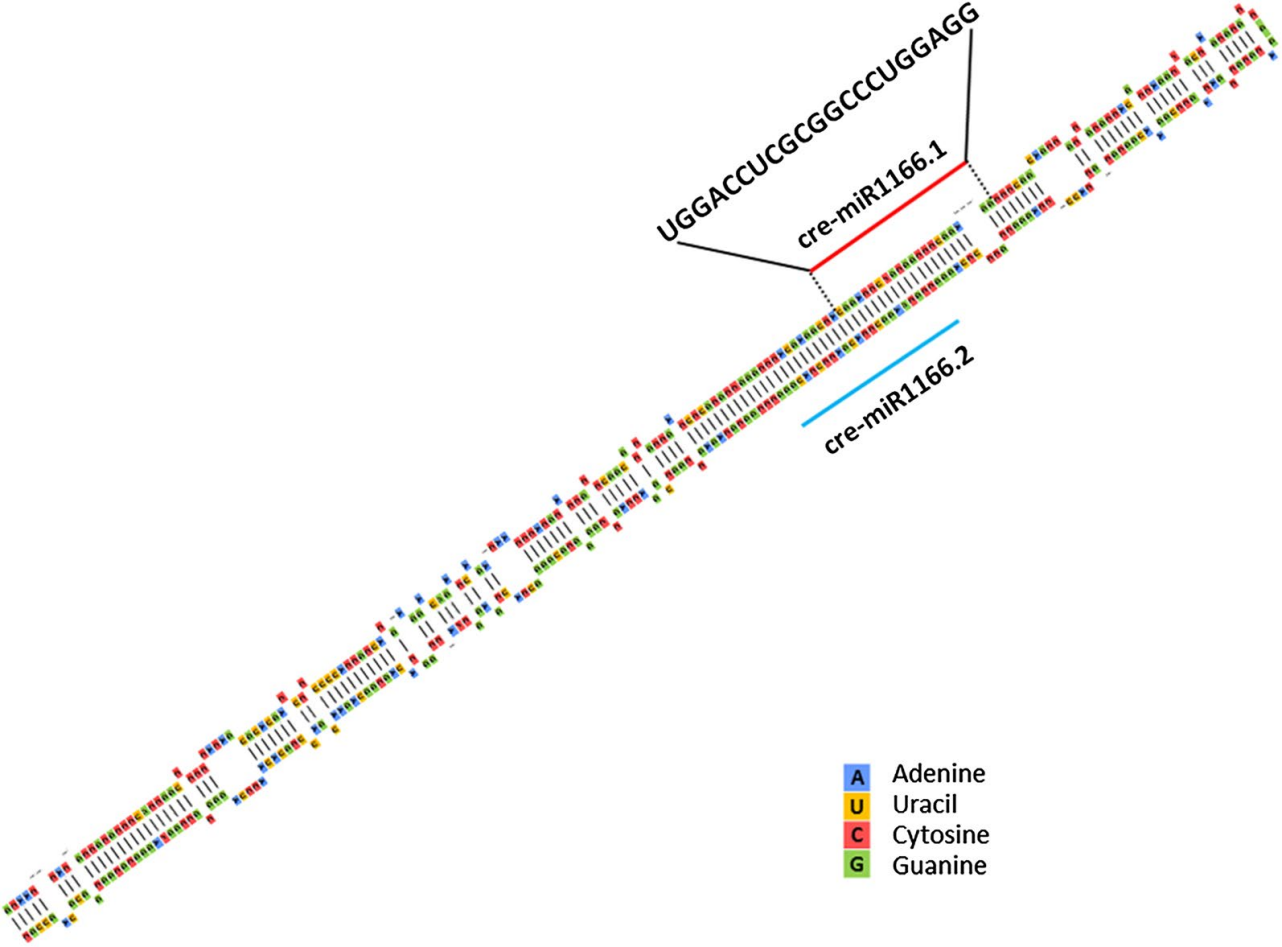
Stem–loop structure of the natural cre-miR1166 precursor. A, U, C, and G indicate adenine, uracil, cytosine, and guanine, respectively. The precursor is 372 nt in length. The red and blue lines mark the position of mature miR1166.1 and mature miR1166.2 within the stem–loop structure, respectively

Cre-miR1166.1 is an endogenous miRNA in *C. reinhardtii* that is cataloged in miRBase (http://www.mirbase.org/) and was also recorded in our previous miRNA sequencing study. The natural miR1166.1 precursor is 372 nt in length, and the 21 nt mature miRNA is located in the 5′ end of the stem–loop structure (Fig. 4). In our miRNA sequencing data, the FPKM (expected number of fragments per kilobase of transcript sequence per million mapped reads) of miR1166.1 was up-regulated from 0.70 to 2.72 after sulfur deprivation (Fig. 1), which suggested a close relationship of this endogenous miRNA with sulfur-deprivation metabolism pathways in *C. reinhardtii*.

### Quantitative real-time PCR analysis of miR1166.1

**Fig. 5:**
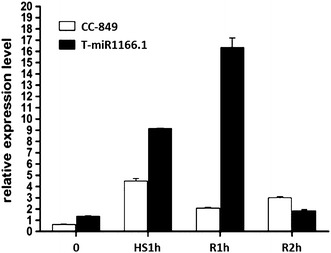
Quantitative real-time PCR analysis of miR1166.1. Expression levels of miR1166.1 in CC-849 and the transgenic strain before and after heat shock and after 1 and 2 h of recovery. *0* untreated controls; *HS1h* heat shock treatment for 1 h; *RT1h and RT2h* recovery at 22 °C for 1 and 2 h after heat treatment, respectively

To test the functionality of the constructed expression vector, the transcription levels of miR1166.1 were detected by qRT-PCR in both CC-849 and transgenic algae at four time points: before heat treatment, after 1 h heat treatment at 40 °C, and after 1 and 2 h of recovery following heat treatment. The primers used in the experiments are listed in Additional file 1: Table S1. miR1166.1 expression was significantly induced in the transgenic strain after heat treatment. miR1166.1 also increased in CC-849, but the levels in the transgenic algae were twice the levels in CC-849. The most significant difference was detected after 1 h of recovery, when the miR1166.1 transcription level in transgenic algae was about nine times greater than that in CC-849 (Fig. 5). These results indicate that mature miR1166.1 was significantly induced in the transgenic strain T-miR1166.1 upon heat induction.

### Enhanced H_2_ production of transgenic algae

**Fig. 6:**
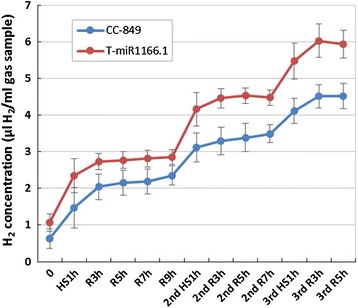
H_2_ production of CC-849 and transgenic algae after heat induction. *0* control; *HS* heat shock; *R* recovery at 22 °C. Gas samples from the headspace of the CC-849 and transgenic algae cultures were measured before and after 1 h heat treatment. The cultures were recovered at 22 °C, and after 3, 5, 7, and 9 h of recovery, H_2_ concentrations were measured. The heat treatment and recovery were implemented three times

To investigate the effect of heat-induced miR1166.1 expression on algal H_2_ production, we designed an experiment involving repeated heat treatment. Algae at exponential phase were treated with 40 °C heat shock, and the H_2_ production levels were detected immediately. The algae were allowed to recover at 22 °C for several hours followed by H_2_ detection. The heat treatment and detections were repeated three times. The raw data for H_2_ production are listed in Additional file 1: Table S2. Each heat shock treatment induced an increase in H_2_ production in both the transgenic and CC-849 algae. However, the transgenic algae maintained a higher H_2_ yield (1.21- to 1.68-fold greater) than CC-849 from the first time point to the end of the experiment (Fig. 6). These results indicate that the overexpression of miR1166.1 can modulate H_2_ production in *C. reinhardtii*.

## Discussion

miRNA functions have been studied extensively in animals and plants, but much has yet to be uncovered about the physiological functions of endogenous miRNAs in the unicellular model organism *C. reinhardtii*. Firstly, there are few studies providing foundational information about non-coding RNAs in this green alga. Additionally, phenotypic assessment of genetic mutants to functionally characterize miRNAs, a powerful approach in plants, is uncommon in microalgae because their phenotypes are difficult to observe. In plants, miRNA regulation affects plant development and morphology, and plant phenotypes can be very informative about miRNA functions and expression levels [29, 37]. In *C. reinhardtii*, a few miRNAs have been confirmed to cleave mRNAs or repress mRNA translation, but no significant phenotypes attributable to miRNA alteration have been reported. The present findings link the phenotype of enhanced hydrogen production with a natural miRNA in the unicellular microalga *C. reinhardtii*, thereby identifying a physiological function of this miRNA.

Sulfur deprivation induces sustained hydrogen production in *C. reinhardtii* [13], and we found that miR1166.1 was significantly up-regulated after sulfur deprivation. We therefore hypothesized that miR1166.1 might be a hydrogen biosynthesis regulating factor. The hydrogen yield data from the heat-inducible miR1166.1 transgenic algal strain confirmed that miR1166.1 enhanced hydrogen biosynthesis and that the high transcription level of miR1166.1 led to the increase in hydrogen yield. This is the first evidence that a natural miRNA in green algae regulates hydrogen photoproduction.

miRNAs in *C. reinhardtii* were considered to play a limited role in responses to nutrient deprivation, including sulfur or phosphate deprivation [40]. In general, the miRNA overexpression transgenic strains usually don’t have lethal phenotype or significantly growth phenotypes according to our experience. In this study, our result shows that endogenous miRNA1166.1 significantly affects H_2_ production in *C. reinhardtii*. We propose that, compared to a signal miRNA like miRNA1166.1, multiple miRNA regulation may have more significant effect. What’s more, the miRNA target prediction in *C. reinhardtii* should be different from that in higher plants and animals, with the rather complicated identification of miRNA target genes, thus the results obtained from predicted targets may not be able to represent the function of miRNAs accurately. More studies on *C. reinhardtii* miRNA functions are still necessary.

We established an efficient heat-inducible system based on the regulatory function of a natural miRNA to control sustained hydrogen production in green algae. The miRNA transcription level (Fig. 5) and hydrogen yield (Fig. 6) showed that the baseline expression of miR1166.1 in the transgenic algal strain was slightly higher than in CC-849. This is probably because the *HSP70*-*RBCS2* promoter can be induced by light as well as heat [4, 32]. However, heat treatment had a stronger effect and could be applied repeatedly to achieve sustained hydrogen production.

We performed qPCR and sequencing to investigate the transcription levels of the miRNA. The result showed that the artificial miRNA precursor had been successfully cleaved to yield the endogenous mature miR1166.1 sequence. Previous studies have demonstrated that the miR1162 backbone is a powerful tool for expressing artificial miRNA sequences [4, 36]. This study further shows that the miR1162 backbone is also applicable for the transgenic expression of natural miRNA sequences.

Target gene prediction of miR1166.1 was performed to investigate the molecular mechanism of this miRNA, but the verification of miRNA target genes is difficult and considerable. Unlike the typically extensive complementarity of miRNAs to their targets in land plants, complementarity to the miRNA seed region is sufficient to induce target gene repression in *C. reinhardtii* [38]. This difference leads to a broader range of miRNA target genes and a higher probability of inaccurate prediction, consequently making miRNA target identification in *C. reinhardtii* more complicated. The investigation of the target genes of endogenous miR1166.1 is currently ongoing in our laboratory.

## Conclusions

miRNAs in the unicellular model organism *C. reinhardtii* were initially reported in 2007, and there have been a few studies about the functional mechanisms of miRNAs in this species. However, the present study is the first to identify the physiological function of a natural miRNA in *C. reinhardtii*. Moreover, natural miR1166.1 regulates hydrogen photoproduction, which is considered a promising strategy for solving energy problems. We previously found that an artificial miRNA targeting *OEE2* could enhance hydrogen production; here, we demonstrated that a natural miRNA could also be a hydrogen photoproduction regulator. The findings may provide a novel approach for improving hydrogen production without medium replacement.

## Additional files

**Additional file 1: Table S1.** Primers used in the experiments. **Table S2.** Total H_2_ yield of CC-849 and T-miRNA1166.1 transgenic algae before and after heat induction.

**Additional file 2: Figure S1.** Verification of transgenic algae by genomic DNA PCR. Target bands are 593 bp in length.

**Additional file 3: Figure S2.** H_2_ concentration of T-miR1150 and CC-849 under heat treatment.

**Additional file 4: Figure S3.** H_2_ concentration of T-miR1158 and CC-849 under heat treatment.
