# Supplementary material for: An endogenous microRNA (miRNA1166.1) can regulate photobio-H2 production in eukaryotic green alga Chlamydomonas reinhardtii
Source: Biotechnol Biofuels. 2018 May 2;11:126. doi: 10.1186/s13068-018-1126-8 (PMC5930490; doi:10.1186/s13068-018-1126-8)
Supplement: Supplementary file 1 — Additional file 1: Table S1. Primers used in the experiments. Table S2. Total H2 yield of CC-849 and T-miRNA1166.1 transgenic algae before and after heat induction. [file 13068_2018_1126_MOESM1_ESM.docx]

**Additional file**

**Table S1** Primers used in the experiments

| **Name** | Sequence | **Annotation** |
| --- | --- | --- |
| **F-1166.1** | CCGTGGACCTCGCGGCCC | Forward primer for qPCR |
| **F-U4** | CGGCGCAAAAGGCCCGACAGAAAT | Reverse primer for qPCR |
| **RT-1166.1** | GTGCAGGGTCCGAGGTCAGAGCCACCTGGGCAATTTTTTTTTTTCCTCCA | Reverse transcription primer |
| **RT-U4** | GTGCAGGGTCCGAGGTCAGAGCCACCTGGGCAATTTTTTTTTTTATTTCTC | Reverse transcription primer |
| **Universal probe** | CAGAGCCACCTGGGCAATTT | Universal probe for qPCR |
| **Universal R** | CAGTGCAGGGTCCGAGGT | Universal reverse primer for qPCR |

**Table S2** Total H_2_ yield of CC-849 and T-miRNA1166.1 transgenic algae before and after heat induction

|  | **C849-1** | **CC849-2** | **CC849-3** | **1166.1-1** | **1166.1-2** | **1166.1-3** |
| --- | --- | --- | --- | --- | --- | --- |
| **RT3Day 0** | - | 12880821 | 5210913 | 10748080 | 15777682 | 19249220 |
| **HS1** | 12844850 | 31575648 | 18590250 | 25165101 | 34255692 | 41419619 |
| **HS1h+3hRT** | 25110742 | 36144309 | 26232899 | 34657873 | 41058555 | 41552169 |
| **HS1h+5hRT** | 26921646 | 37539826 | 27847821 | 35003212 | 41721621 | 41941255 |
| **HS1h+7hRT** | 29031793 | 38040741 | 26800949 | 35645485 | 41737240 | 43140175 |
| **HS1h+9hRT** | 30810109 | 38982402 | 31005743 | 36691476 | 42575346 | 43143189 |
| **RHS.2+1h** | 39778777 | 52286599 | 41375752 | 51300407 | 60027572 | 67212336 |
| **RHS2+3h** | 42809105 | 54734706 | 43730878 | 58839250 | 64468245 | 67868364 |
| **RHS2+5h** | 43975899 | 56087691 | 44943528 | 60869942 | 65159217 | 68206690 |
| **RHS2+7h** | 47308106 | 54937122 | 47361688 | 60223285 | 64141901 | 67567175 |
| **RRHS+1h** | 55229979 | 65745826 | 55626562 | 73160414 | 73271250 | 88107577 |
| **RRHS+3h** | 63571420 | 70417472 | 59416399 | 83578366 | 79921578 | 94950049 |
| **RRHS+5h** | 62886829 | 71224102 | 59614804 | 82762702 | 79557685 | 92284446 |

The numbers in the table represent the H_2_ peak area.
