# Supplementary figures and images for: An endogenous microRNA (miRNA1166.1) can regulate photobio-H2 production in eukaryotic green alga Chlamydomonas reinhardtii
Source: Biotechnol Biofuels. 2018 May 2;11:126. doi: 10.1186/s13068-018-1126-8 (PMC5930490; doi:10.1186/s13068-018-1126-8)

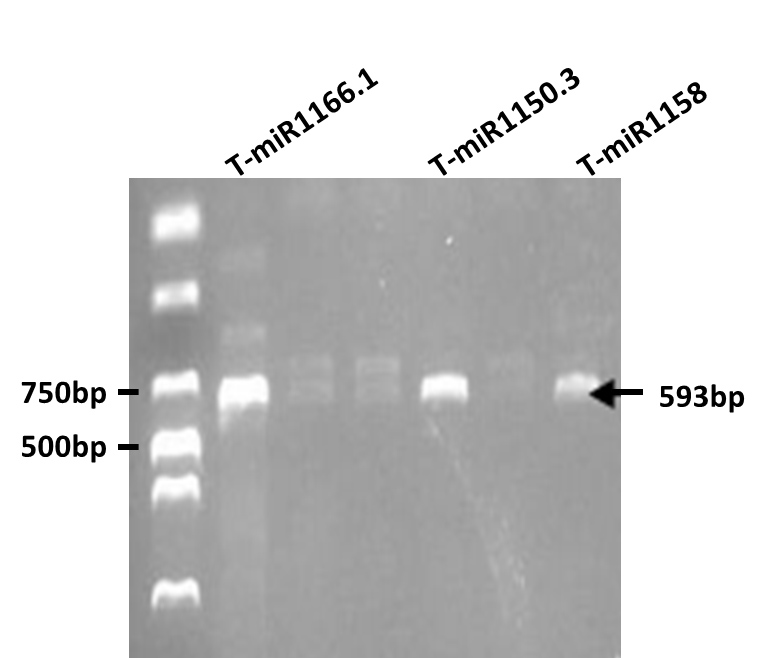

Supplement: Supplementary file 2 — Additional file 2: Figure S1. Verification of transgenic algae by genomic DNA PCR. Target bands are 593 bp in length. [file 13068_2018_1126_MOESM2_ESM.jpg]

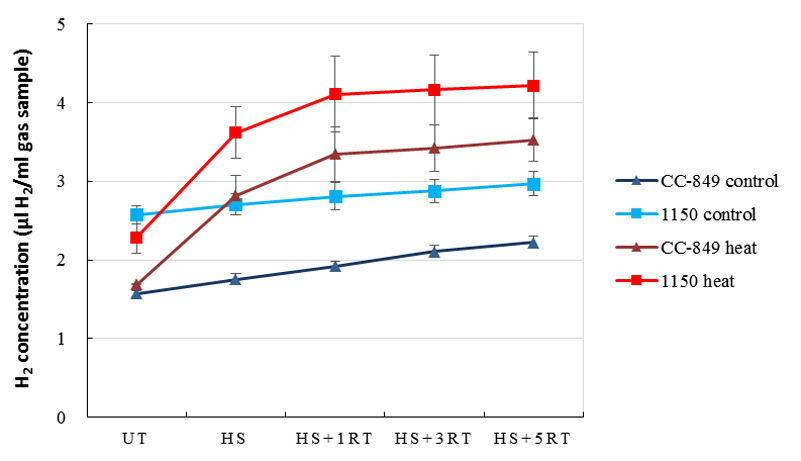

Supplement: Supplementary file 3 — Additional file 3: Figure S2. H2 concentration of T-miR1150 and CC-849 under heat treatment. [file 13068_2018_1126_MOESM3_ESM.jpg]

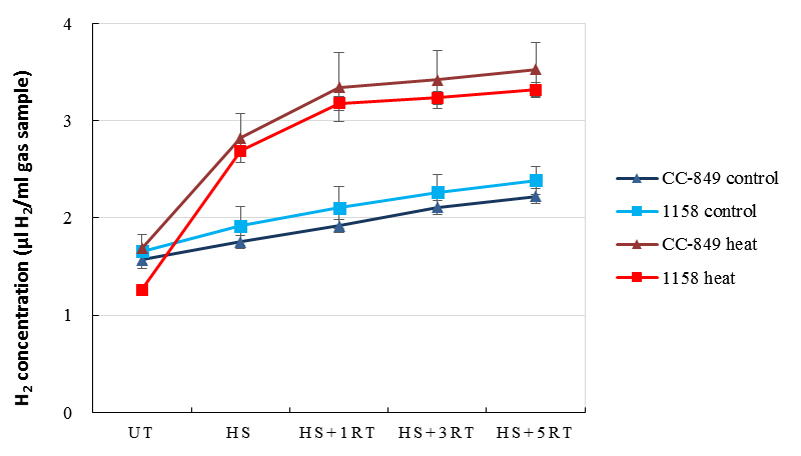

Supplement: Supplementary file 4 — Additional file 4: Figure S3. H2 concentration of T-miR1158 and CC-849 under heat treatment. [file 13068_2018_1126_MOESM4_ESM.jpg]
